# Interval probabilistic neural network

**DOI:** 10.1007/s00521-015-2109-3

**Published:** 2015-11-21

**Authors:** Piotr A. Kowalski, Piotr Kulczycki

**Affiliations:** 1 Systems Research Institute, Polish Academy of Sciences, ul. Newelska 6, 01-447 Warsaw, Poland; 2 Faculty of Physics and Applied Computer Science, AGH University of Science and Technology, al. A. Mickiewicza 30, 30-059 Cracow, Poland

**Keywords:** Neural network, Interval probabilistic neural network, Data analysis, Classification, Interval data, Imprecise information, Numerical simulation

## Abstract

Automated classification systems have allowed for the rapid development of exploratory data analysis. Such systems increase the independence of human intervention in obtaining the analysis results, especially when inaccurate information is under consideration. The aim of this paper is to present a novel approach, a neural networking, for use in classifying interval information. As presented, neural methodology is a generalization of probabilistic neural network for interval data processing. The simple structure of this neural classification algorithm makes it applicable for research purposes. The procedure is based on the Bayes approach, ensuring minimal potential losses with regard to that which comes about through classification errors. In this article, the topological structure of the network and the learning process are described in detail. Of note, the correctness of the procedure proposed here has been verified by way of numerical tests. These tests include examples of both synthetic data, as well as benchmark instances. The results of numerical verification, carried out for different shapes of data sets, as well as a comparative analysis with other methods of similar conditioning, have validated both the concept presented here and its positive features.

## Introduction

In the last ten years, interest has notably arisen in probabilistic neural network (PNN) [38] methodology. This statement can be affirmed through perusing its application within a large number of recent scientific articles. PNN methodology is often used particularly in classification tasks, and it has been applied successfully in solving problems related to email security enhancement [40], most certainly within the intrusion detection systems [41]. This concept for easing the classification task has also been applied in medicine, especially for detecting resistivity to antibiotics [5], as well as for diagnosing hepatitis [2] and in gaining solutions to other medical problems [12, 29]. Moreover, the method under consideration enables the solving of different problems related to pattern recognition tasks, among these being biometric speech and speaker recognition [9, 30], in addition to signature verification [28]. The PNNs are also constructed to gain solutions within marine administration problems such as effective ship identification [1]. Another group of studies wherein this neural network is applied is in chemical and environmental sciences [14, 39, 46].

In many applications, the conceptualization of PNN has, however, changed. Where once it was associated with the working with the large number of neurons that are derived from a large number of pattern samples within the first layer, now it is often used in the structuring of the network so as to reduce the quantity of hidden neurons in the second layer. This is especially evident in research on data for medical problems [23], which usually have a significant amount of reference examples. Another type of PNN modification is seen in the adaptation of neural operations to other data types. An illustrative example of this is the PNN adjustment for nonlinear time series analysis [44]. Furthermore, during the development of methods exploiting PNN, still more type changes in the network were introduced. These assumed a reduction in neural structure that was engendered by the selection of good training sets. Moreover, in this case, principal component analysis was used to reduce the computational complexity of the investigated network [3].

Due to current dynamic development in exploratory data analysis, a widening interest in interval analysis can be seen. Herein, the fundamental application of interval analysis is to ensure a suitable precision quality for numerical calculations. A very important advantage of this action is the fact that it has a formal mathematical algebra [11], but the biggest advantage of this approach is its ability to describe inaccuracies in the simplest possible way. In other methods, such as probability [10] or fuzzy logic [13], to describe the uncertainty, a much greater amount of information is required. Here, only one available set of information about an investigated quantity *x* can be built upon, providing that it is included in the interval sample $[\underline{x},\overline{x}]$. Moreover, in many application tasks, this simple assumption is absolutely sufficient. What is more, in a simple way, the use of interval analysis can be extended to a multidimensional case. Consequently, the algorithm is very easy to interpret. Furthermore, the calculation complexity is reduced with respect to other similar methods used for uncertainty modeling.

In this paper, the generalized probabilistic neural network to interval data is introduced. The employment of this requires a change in the structure of the network through the introduction of special transfer functions in the neurons located inside the hidden layer. This classification concept is based on the Bayes approach, which insures a minimum of potential losses occurring through misclassifications. For this concept, the PNN procedure was employed, thereby freeing the above method from arbitrary assumption regarding pattern class. Thus, their identification becomes an integral part of the proposed algorithm. The term “interval probabilistic neural network” (IPNN) is the name put forward for such a created structure.

In the procedure for classifying based on IPNN, the elements of pattern sets are given as precise data (e.g., deterministic or shape), but work on the tested element comes about by considering imprecise information as being an interval or interval vector (in multidimensional cases):

1 $$\begin{aligned} \left[ \begin{array}{c} {[}\underline{x}_1,\overline{x}_1] \\ {[}\underline{x}_2,\overline{x}_2] \\ \dots \\ {[}\underline{x}_n,\overline{x}_n] \end{array} \right] , \end{aligned}$$

where $\underline{x_k}\leqslant \overline{x_k}$ for $k=1,2,\ldots ,n$, is represented.

Here, the pattern sample is considered to be composed of actual, precisely measured quantities that can be traded, while the intervals are classified as representing uncertainties and imprecisions in plans, estimations, or measurements that are hard to remove. In particular, pattern sets may consist of accurate measurements (in which errors are practically ignored), while the classified interval contains measurements taken by way of another, much less accurate apparatus or which were carried out in much worse conditions. Another example of the application of this kind of classification is its use in treating precise data as actual information from the past, e.g., temperature or currency exchange rates, while the classified element represents a prognosis, which by nature is limited in precision. In these cases, the cardinality of the pattern data is often very large. Therefore, it is advisable to use an intelligent method to reduce the unnecessary (e.g., outliers elements) or redundant components inside the pattern sets.

This paper is organized as follows. The first part of the paper is devoted to a description of PNN methodology. Of note is the laying out of how the characterization of kernel density estimators (KDE) with the smoothing parameter calculation algorithm and procedure for its modification takes place. Afterwards, the subsequent subsections concentrate on the neural representation of the KDE as a PNN. In the following part of this article, the extension of the PNN theory to the notion of it being an interval probabilistic neural network is put forward. The full algorithm for neural classification of imprecise information is the main subject of the next section, and the numerical verification for the proposed methodology is to be shown in Sect. 6. Finally, some concluding remarks with respect to the presented approach are set out.

The preliminary version of this investigation as a conference short paper, by Kowalski and Kulczycki [19], has already been presented.

## Probabilistic neural network

### Kernel density estimator

In most practical data exploration tasks, the probability density function *f* of a given sample is multimodal and can be mapped into a function of any typical distribution. Therefore, methods of nonparametric density estimation which do not need any assumptions on the distribution type are in common use. The kernel density estimation (KDE) is one of the classical techniques with such property [36].

Consider a *n*-dimensional random variable, with a distribution having the density *f*. A kernel estimator of this function $\hat{f}:\mathbb {R}^n \rightarrow [0, \infty )$ based on the *m*-elements data sample $x_1, x_2, \dots , x_m$ can be defined as:

2 $$\begin{aligned} \hat{f}(x)=\frac{1}{mh^{n}}\sum _{i=1}^{m}{K\left( \frac{x-x_{i}}{h}\right) }. \end{aligned}$$

The positive coefficient *h* is called a “smoothing parameter,” while the measurable function $K(x):\mathbb {R} \rightarrow [0, \infty )$, of unit integral, symmetrical with respect to zero, and having a weak global maximum at this point, is called a “kernel.” In this approach, the Cauchy kernel

3 $$\begin{aligned} K(x)=2/{\pi (x^2+1)^2} \end{aligned}$$

will be used. In the multidimensional case, this can be generalized to the product kernel notation:

4 $$\begin{aligned} K(x)=K(x_1, x_2,\dots , x_n)^{\rm T}=\mathcal {K}(x_1)\cdot \mathcal {K}(x_2)\cdot \ldots \cdot \mathcal {K}(x_n), \end{aligned}$$

where $\mathcal {K}$ constitutes the one-dimensional Cauchy kernel given above. As a result, the smoothing parameter takes the form of a vector $(h_1, h_2, \dots , h_n)$. This can be easily obtained using automatic smoothing selection procedures, i.e., the plug-in method or the cross-validation procedure.

**Fig. 1:**
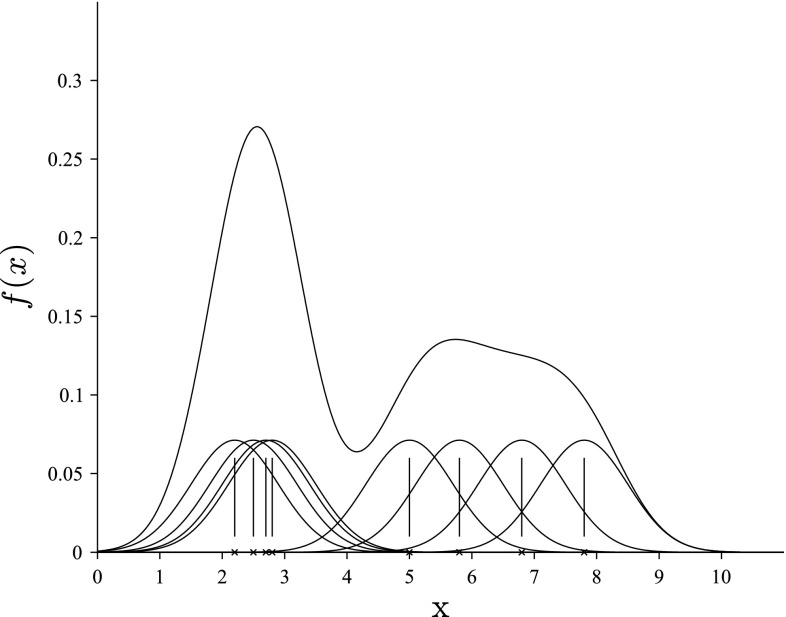
The KDE function

The interpretation of the above definition is illustrated in Fig. 1 for a one-dimensional ($n=1$) random variable. In the case of the single realization $x_i$, the function *K* (transposed along the vector $x_i$ and scaled by the coefficient *h*) represents the approximation of distribution of the random variable *X* having obtained the value $x_i$. In this example, the data sample contains eight elements ($m=8$), which are marked using *x* on the horizontal axis.

Of note is that the quality of the estimation of function (2) depends upon both the use of an appropriate smoothing parameter, as well as on the application of the procedures for its modification. These tasks will be presented in detail in the following subsections.

More in-depth information about the practical issues of KDE methods, as well as usage examples, can be found in cited references [20, 36, 43].

### Smoothing parameter

In this subsection, presentation will be made of the algorithm for the calculation of the smoothing coefficients. Although it is a known algorithm, the authors of this paper wish to provide a limited discussion, as doing so complements the full article, and enables the methodology presented herein to be employed without recourse to additional bibliographic sources. This, the authors feel, is of particular importance, as the described interval neural network procedure constitutes the complete (entire) algorithm.

A very important consideration that must be thought out so as to achieve a high quality estimation is making an appropriate choice of smoothing coefficient. If this is too large, the value brings about excessive flattening of the kernel estimator function, while a too little value results in the formation of many peaks. Most known algorithms for the generation of an optimal value of this coefficient are based on the minimization of mean-square-error MISE. Moreover, the plug-in algorithm and cross-validation procedure are the most common methods used for parameter *h* calculation. What is more, the cross-validation method is recommended for application in the case of multidimensional variables, while a radial type of kernel function is used. Due to the use of the kernel product conception, in this work, the plug-in algorithm will be presented in-depth.

The optimal value of the smoothing parameter may be obtained, as mentioned above, by minimizing the following equation (typical for KDE methods) with respect to the argument *h* (for details, see [43, Section 3.4])

5 $$\begin{aligned} MISE(\hat{f})=\frac{1}{mh^n}R(K)+\frac{1}{4}h^4P(K)^2R(\nabla ^2f). \end{aligned}$$

Equation (5) is a consequence of the generally applicable accuracy assessment of estimation based on the minimization of the integrated average quadratic performance index MISE:

6 $$\begin{aligned} MISE(\hat{f})=E\left( \int _{R^n}\left[ \hat{f}(x)+f(x)\right] ^2 \hbox {d}x \right) , \end{aligned}$$

where *E* denotes the expected value of the distribution of the *n*-dimensional random variable *X*. Furthermore, for complementarity, the following describes the individual components of formula (5):

7 $$\begin{aligned} R(K)= \int _{R^n}K(x)^2 \hbox {d}x , \end{aligned}$$

8 $$\begin{aligned} P(K)= \int _{R^n}x^{\rm T} x K(x) \hbox {d}x , \end{aligned}$$

9 $$\begin{aligned} R(\nabla ^2f)= \int _{R^n}\left( \nabla ^2f(x)\right) ^2 \hbox {d}x , \end{aligned}$$

and, finally, taking into account the following notation,

10 $$\begin{aligned} \nabla ^2f(x)=\sum _{i=1}^{n}\frac{\partial ^2f(x)}{\partial x_i^2} . \end{aligned}$$

On the basis of the above formulas, the optimum value *h* can be obtained as

11 $$\begin{aligned} h=\left[ \frac{nR(K)}{mP(K)^2R(\nabla ^2f)} ]\right] ^\frac{1}{n+4}. \end{aligned}$$

Therefore, the value of criterion (5) for *h* as taken from formula (11) is given as

12 $$\begin{aligned} MISE(\hat{f})=\frac{n+4}{4n}C(K)\left[ \frac{n^4R(\nabla ^2f)^n}{m^4} \right] ^\frac{1}{n+4} , \end{aligned}$$

where

13 $$\begin{aligned} C(K)=\left[ R(K)^4P(K)^{2n}\right] ^\frac{1}{n+4}. \end{aligned}$$

In the present study, due to the use of the concept of kernel product, it is sufficient to present the case of a one-dimensional plug-in algorithm. This method is characterized by its simplicity, as well as by the accuracy of the results.

For a one-dimensional case, formula (11) consequently takes the following form

14 $$\begin{aligned} h=\left[ \frac{R(\mathcal {K})}{mP(\mathcal {K})^2R(f'')}\right] ^\frac{1}{5}. \end{aligned}$$

The right part of formula (14) depends primarily on the expression $R(f'')$. For the second derivative $f''$ estimate, another estimator based on any kernel $K_1$ can be used. The full mathematical description of this algorithm can be found in [43, Section 3.6].

As this method is an approximation algorithm, it is necessary to approach it iteratively. For the purpose of this study, three iterations $(q=3)$ of the procedure were established, and numerical verification tests have confirmed the validity of this assumption. Thus, the plug-in method consists of accepting the following steps:

*Step 1* Estimate $\psi _{10}$ by using the normal scale estimate $\psi _{10}^{NS}$

15 $$\begin{aligned} \psi _{10}^{NS}=\frac{-945}{64\pi ^{\frac{1}{2}}\hat{\sigma }^{11}}. \end{aligned}$$

where $\hat{\sigma }$ denotes the estimate of data set standard deviation. This formula is treated as a first approximation of $\psi$, and it is based on the assumption that the data are characterized by variance $\sigma ^2$ [43].

*Step 2* Estimate $g_{1}$ by using estimator $\psi _{10}^{NS}$

16 $$\begin{aligned} g_{1}=\left( \frac{-2K_1^{(8)}(0)}{mP(K_1)\psi _{10}^{NS}}\right) ^\frac{1}{11}, \end{aligned}$$

*Step 3* Estimate $g_{2}$ by using estimator $\hat{\psi }_{8}(g_1)$

17 $$\begin{aligned} g_{2}=\left( \frac{-2K_1^{(6)}(0)}{mP(K_1)\hat{\psi }_{8}(g_1)}\right) ^\frac{1}{9}, \end{aligned}$$

*Step 4* Estimate $g_{3}$ by using estimator $\hat{\psi }_{6}(g_2)$

18 $$\begin{aligned} g_{3}=\left( \frac{-2K_1^{(4)}(0)}{mP(K_1)\hat{\psi }_{6}(g_2)}\right) ^\frac{1}{7}, \end{aligned}$$

*Step 5* And, finally, the smoothing parameter can be obtained as

19 $$\begin{aligned} h=\left( \frac{R(\mathcal {K})}{mP(\mathcal {K})^2\psi _{4}(g_3)}\right) ^\frac{1}{5}. \end{aligned}$$

All the above are applied in the following notation:

20 $$\begin{aligned} \hat{\psi }_{r}(g_q) = \frac{1}{m^2g_q^{r+1}}\sum _{i=1}^{m} \sum _{j=1}^{m}K_1^{(r)}\left( \frac{x_i-x_j}{g_q}\right) . \end{aligned}$$

In Eqs. (16)–(19), we have only even values of indices for estimators $\psi _i$ as a consequence of the fact that we can estimate $\psi _i$ by way of using another kernel estimator. Therein, optimal bandwidth depends on $\psi _{i+2}$. In the proposed case, three iterations of algorithm were introduced; thus, the parameters i were increased by the value 2, starting from value 6 and ending on 10. Here, the following assumptions were made: Initially, the kernel function $K_1$ is k times differentiable, while the even derivatives fulfill the following conditions:

21 $$\begin{aligned} K_1^{(4)}(0)> 0 \end{aligned}$$

22 $$\begin{aligned} K_1^{(6)}(0)< 0 \end{aligned}$$

23 $$\begin{aligned} K_1^{(8)}(0)> 0. \end{aligned}$$

The presentation of an iterative algorithm for calculating the smoothing parameters is characterized by the simplicity of its use within the numerical calculations. If necessary, this algorithm can easily be extended by increasing the number of iterations (i.e., $q>3$).

### Modification of the smoothing parameter

For the better similarity of the estimation of PNN to the real data distribution, a procedure to modify the smoothing parameter should be applied. The main purpose of so doing is to introduce the modification coefficients for smoothing parameters which correspond with each element of the pattern set. The complete algorithm is as follows:

*Step 1* Precomputing the smoothing parameter using the plug-in method and the calculation of the kernel estimator quantities $\hat{f}_{*}(x_i)$ in their basic form (2), for each element $x_i$ contained in the pattern set (for $i=1,2,\ldots ,m$).

*Step 2* For every $x_i$, the modification parameter $s_i$ should be obtained, according to the following formula:

24 $$\begin{aligned} s_i= \left( {\frac{\hat{f}_{*}(x_i)}{\tilde{s}}}\right) ^{-c} \end{aligned}$$

where $\tilde{s}$ is geometrical mean of the estimator quantities $\hat{f}_{*}(x_i)$ for $i=1,2,\ldots ,m$. The constant $c \ge 0$ is referred to as the “modification intensity.” The case $c = 0$, implying in consequence, $s_i \equiv 1$, determines the lack of smoothing parameter modification, whereas, together with an increase in the value *c*, its intensity increases. Corollaries resulting from the mean-square criterion primarily point to the value:

25 $$\begin{aligned} c=0.5 \end{aligned}$$

*Step 3* Finally, the kernel estimator formula takes the form as follows:

26 $$\begin{aligned} \hat{f}(x) = \frac{1}{mh^{n}}\sum _{i=1}^{m}{\frac{1}{s_i^n}K \left( \frac{x-x_{i}}{hs_i}\right) }. \end{aligned}$$

These coefficients put more personalizations of subsequent elements of the data set in the construction of the estimator function, by changing the shape of the kernels. In the areas where elements of the sample set are dense, for the elements $x_i$, it is true that $f_{*}(x)$ is greater then $s_i$, and therefore, as a result of formula (24), also $s_i<1$. This leads to a narrowing of the kernels assigned to them, which, in turn, allows for better characterization of specific properties of distribution. In opposite cases, in the areas where the elements of the data set are sparse, $f_{*}(x)$ will be lower then $s_i$, and consequently, $s_i>1$. This induces a “flattening” and thus is advantageous to the estimation quality. Moreover, it provides additional smoothing of the kernel estimator within the peripheral regions (primarily, the so-called tails) of distribution. In classification tasks, this is very important, and it places this especially near the boundaries of specific classes, which makes this procedure particularly useful.

### KDE: neural representation

**Fig. 2:**
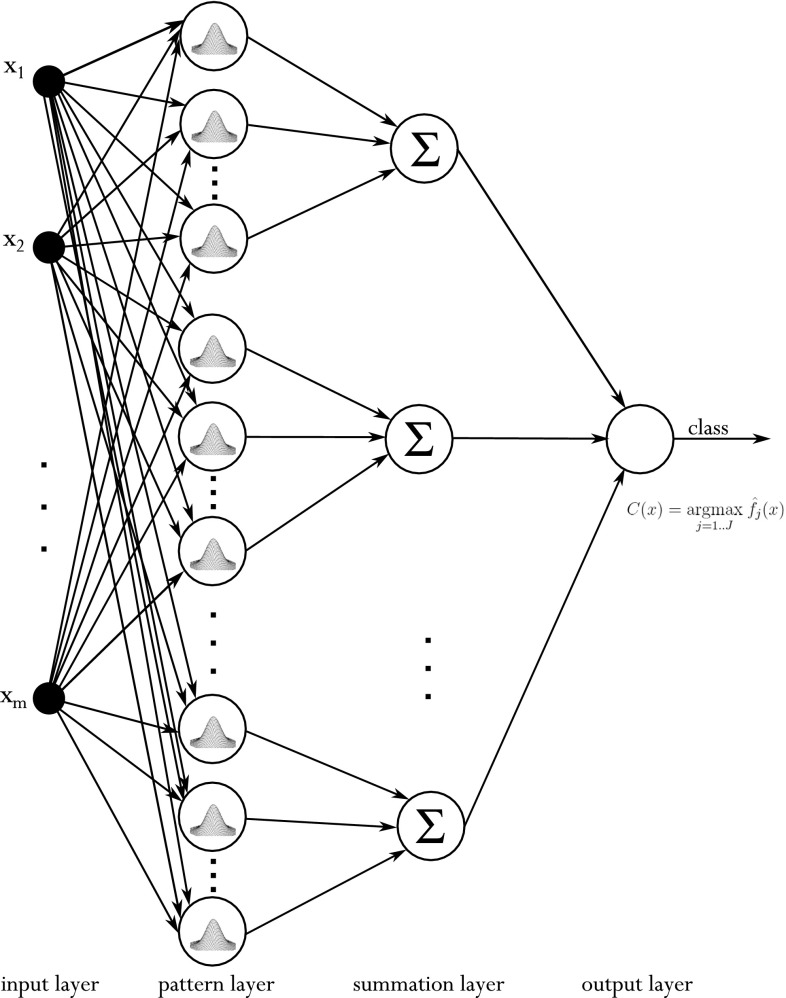
The basic architecture of the PNN

Very often, a PNN, being a special type of Radial Neural Network, is based on the KDE methodology. This type of neural network is employed in regression [34], prediction [40], and classification [23] tasks, but it is also used for nonlinear time series analysis [44]. For providing solutions to classification problems, the standard architecture of this type of network is depicted in Fig. 2.

In this structure, four layers of the network can be seen. The first, the input layer, is represented by the attributes of input data *x*. The subsequent is called the “pattern layer.” This part of the neural structure contains the elements of the learning sample. These are placed in separate (for each class) parts of the network. The kernel transformation (3)–(4) acting as an activation function within the hidden layer is then applied here. The third layer is called the summation layer, and it is composed of *J* (number of classes under investigation) neurons, where each one generates the signals only for the particular pattern which belongs to the *j*-th class, according to formula (2). Finally, the output layer consists of only one neuron. This provides an estimation of the class of investigated element *x*. It is based on the outputs of all the summation layer neurons (in accordance with Bayes theorem). Here, all input signals represent the value of the KDE in each class for *x* input vector. According to Bayes theorem, the new tested element is then assigned to the class for which the input signal is the greatest (27). The above task is accomplished by way of this last output neuron.

27 $$\begin{aligned} C(x) = \underset{j=1,\ldots ,J}{\mathrm {argmax}} \, \hat{f}_j(x) \end{aligned}$$

where *C*(*x*) denotes the predicted class of the new tested element *x*.

The case in which for two or more classes, that the discovered value $\hat{f}_j(x)$ would have equivalence, we think, is very unlikely. What is more, in computer-assisted calculation practices, that this situation could arise when undertaking real data type processing, we feel, is of practical impossibility.

### Training process

In the PNN, regarding the learning process, initially, a topological structure should be designed which takes into account the number of classes under consideration, as well as the cardinality of the learning samples. The first step in so doing presupposes a coupling of the training samples to the second layer of the network. Therefore, the number of hidden neurons is assumed to be equal to the size of sample sets in each class. The second step of the training procedure is connected with applying the algorithm for calculating the smoothing parameters $(h_i)$ and for determining the modification coefficients $(s_j)$. Both procedures in Sects. (2.2) and (2.3) are then introduced. After the placement of these parameters within the network structure, a classifier is obtained, which is ready to operate.

## Extension of PNN for interval data

Very often, a PNN can be treated as a neural realization of the involved KDEs [34, 37]. By means of the form of KDE presented in this paper, the classification procedure can also be interpreted as being a natural generalization of the PNN. In contrast to classical neural networks (e.g., MLP) as commonly used within probabilistic networks, there is no problem in the selection of optimal connection weights. Moreover, the form of KDE brings about a situation wherein that individual components of the learning sample are treated as being neural structure elements. This is the significant advantage of the training procedure in this type of network. However, in the literature put forward with regard to application tasks, the issue of setting parameters that have influence upon learning process is practically ignored due to the absence of a suitable mathematical apparatus. The methodology proposed in this article, the stated issues of optimal selection of these parameters, and the introduction of additional procedures have been carefully considered and analyzed in the previous chapter.

### Structure of the interval probabilistic neural network

**Fig. 3:**
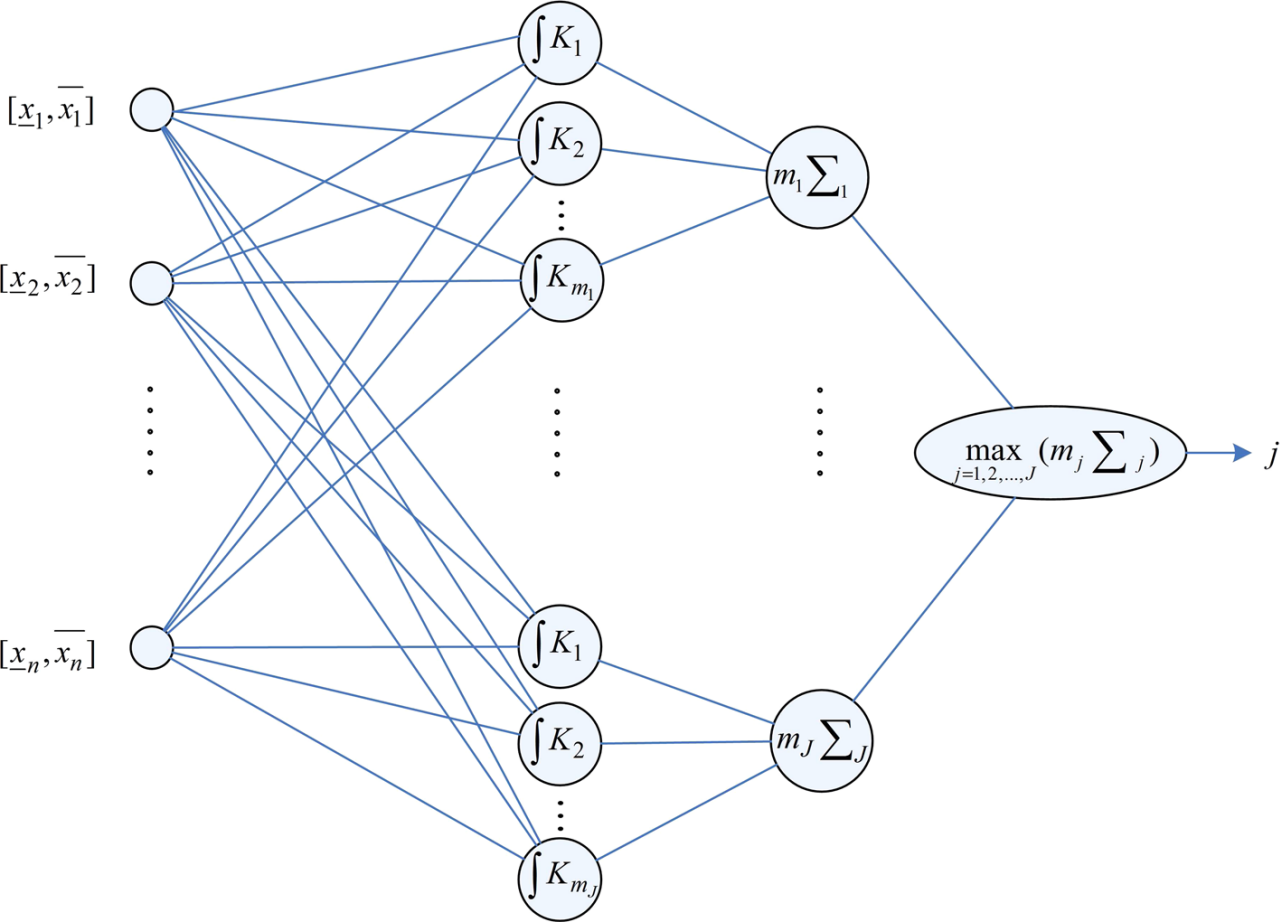
Structure of the interval probabilistic neural network

In this section, a new way of structuring IPNN will be introduced. The form of IPNN structure takes is based on Specht’s probabilistic network [38], but it includes several new elements which enable us to better classify interval information. Figure 3 reveals a topological scheme of a generalized PNN for interval-type information processing that is particularly useful for tasks involving classification.

In the created network, four layers can be distinguished. The first is the input layer, which is of the size of *n*, wherein the inputs correspond to particular features of the classified interval item. The next layer is a subset of neurons representing successive patterns of classes given as precise data. Each of these consists of an adequate number of neurons which are tasked with performing integration operations (more details associated with this novelty of element will be presented in the next subsection). The third is a set of neurons which summarize the signals generated within a pattern class area, and which then multiply the resulting value by the cardinality of the considered class. The final single neuron, located in the output layer, designates the greatest value of received signal, hereby determining the final outcome of the classification problem according to formula (27), doing so in an analogous manner that is similar to classical PNN cases.

### Mathematical model

Now, denote $\hat{f}_1, \hat{f}_2, \dots , \hat{f}_J$ as the KDEs that are associated with the learning data samples representing every considered class. According to the Bayes approach, which ensures a minimum of potential losses due to error of classification, if sample sizes $m_1, m_2, \dots , m_J$ are corresponding to the “frequency” of occurrence of the elements from each class, then the new tested item $\widetilde{x}\in {\mathbb {R}}$ belongs to the class for which the value $m_1\hat{f}_1(\widetilde{x}), m_2\hat{f}_2(\widetilde{x}), \dots , m_J\hat{f}_J(\widetilde{x})$ is the largest. Therefore, in a situation wherein information is given by the interval $[\underline{x},\overline{x}]$, the tested element belongs to the class for which the value

28 $$\begin{aligned} \frac{m_1}{\overline{x}-\underline{x}} \int _{\underline{x}}^{\overline{x}} \hat{f}_1(x) \, \hbox {d}x,\; \frac{m_2}{\overline{x}-\underline{x}} \int _{\underline{x}}^{\overline{x}} \hat{f}_2(x) \, \hbox {d}x, \dots ,\, \frac{m_J}{\overline{x}-\underline{x}} \int _{\underline{x}}^{\overline{x}} \hat{f}_J(x) \, \hbox {d}x \end{aligned}$$

is the largest. In this formula, the positive constants $1/(\overline{x}-\underline{x})$ can be omitted, as they are negligible for the optimization problem under consideration. Hence, the formula can be presented in the following form:

29 $$\begin{aligned} m_1 \int _{\underline{x}}^{\overline{x}} \hat{f}_1(x) \, \hbox {d}x,\; m_2\int _{\underline{x}}^{\overline{x}} \hat{f}_2(x) \, \hbox {d}x,\dots ,\, m_J \int _{\underline{x}}^{\overline{x}} \hat{f}_J(x) \, \hbox {d}x \end{aligned}$$

Moreover, for any $j = 1, 2,\ldots , J$ one can note

30 $$\begin{aligned} \int _{\underline{x}}^{\overline{x}} \hat{f}(x) \, \hbox {d}x=\hat{F}(\overline{x})-\hat{F}(\underline{x}), \end{aligned}$$

where $\hat{F}$ means the primitive of the function $\hat{f}$. For the kernel (3) used here, the following formula has been calculated analytically (here, some constant values can also be omitted):

31 $$\begin{aligned} \hat{F}(x)=\sum _{i=1}^{m} \left[ \frac{(x^2-2xx_i+x^2_i+h^2)\arctan (\frac{x-x_i}{h})+h(x-x_i)}{x^2-2xx_i+x^2_i+h^2}+\frac{\pi }{2}\right] . \end{aligned}$$

The above formula, useful within the additional procedures often employed when kernel estimators are applied practically, is easily generalized. The main goal of this is to generate the possibility of modifying the smoothing parameter procedure [43]. Consequentially, in this case, equation (31) takes the following form:

32 $$\begin{aligned} \hat{F}(x)=\sum _{i=1}^{m} \left[ \frac{(x^2-2xx_i+x^2_i+h^2s_i^2)\arctan (\frac{x-x_i}{s_ih})+hs_i(x-x_i)}{x^2-2xx_i+x^2_i+h^2s_i^2}+\frac{\pi }{2}\right] . \end{aligned}$$

Finally, formulas (29)–(32) define a complete algorithm of classification in the one-dimensional case.

### Multidimensional classification

In the multidimensional case, when information is given by the interval vector

33 $$\begin{aligned} \left[ [\underline{x}_1,\overline{x}_1], [\underline{x}_2,\overline{x}_2],\dots ,[\underline{x}_n,\overline{x}_n] \right] ^{\rm T} , \end{aligned}$$

the new element belongs to the class for which the value

34 $$\begin{aligned} m_1 \int _{E} \hat{f}_1(x) \, \hbox {d}x,\; m_2\int _{E} \hat{f}_2(x) \, \hbox {d}x,\dots ,\, m_J \int _{E} \hat{f}_J(x) \, \hbox {d}x , \end{aligned}$$

where $E = [\underline{x}_1, \overline{x}_1] \times [\underline{x}_2 \times \overline{x}_2]\times \dots \times [\underline{x}_n, \overline{x}_n]$ is the largest. According to the properties of the product kernel used here, calculations of the values of the *n*-dimensional integrals stated above can be decomposed to the *n* independent one-dimensional tasks. This is because of the dependence:

35 $$\begin{aligned} \int _{E}K(x) \, \hbox {d}x=[\mathcal {J}(\overline{x}_1)-\mathcal {J}(\underline{x}_1)] [\mathcal {J}(\overline{x}_2)-\mathcal {J}(\underline{x}_2)] \dots [\mathcal {J}(\overline{x}_n)-\mathcal {J}(\underline{x}_n)] , \end{aligned}$$

where $\mathcal {J}$ is the primitive function of the (one-dimensional) kernel $\mathcal {K}$. Taking into account the formulas (30)–(32) all used for obtaining the one-dimensional case, this formula completes the algorithm for classifying interval information, particularly for the multidimensional case. Additional information regarding Bayes method and its applications can be found in the work [18].

## Complete neural algorithm for the classification of interval information

**Fig. 4:**
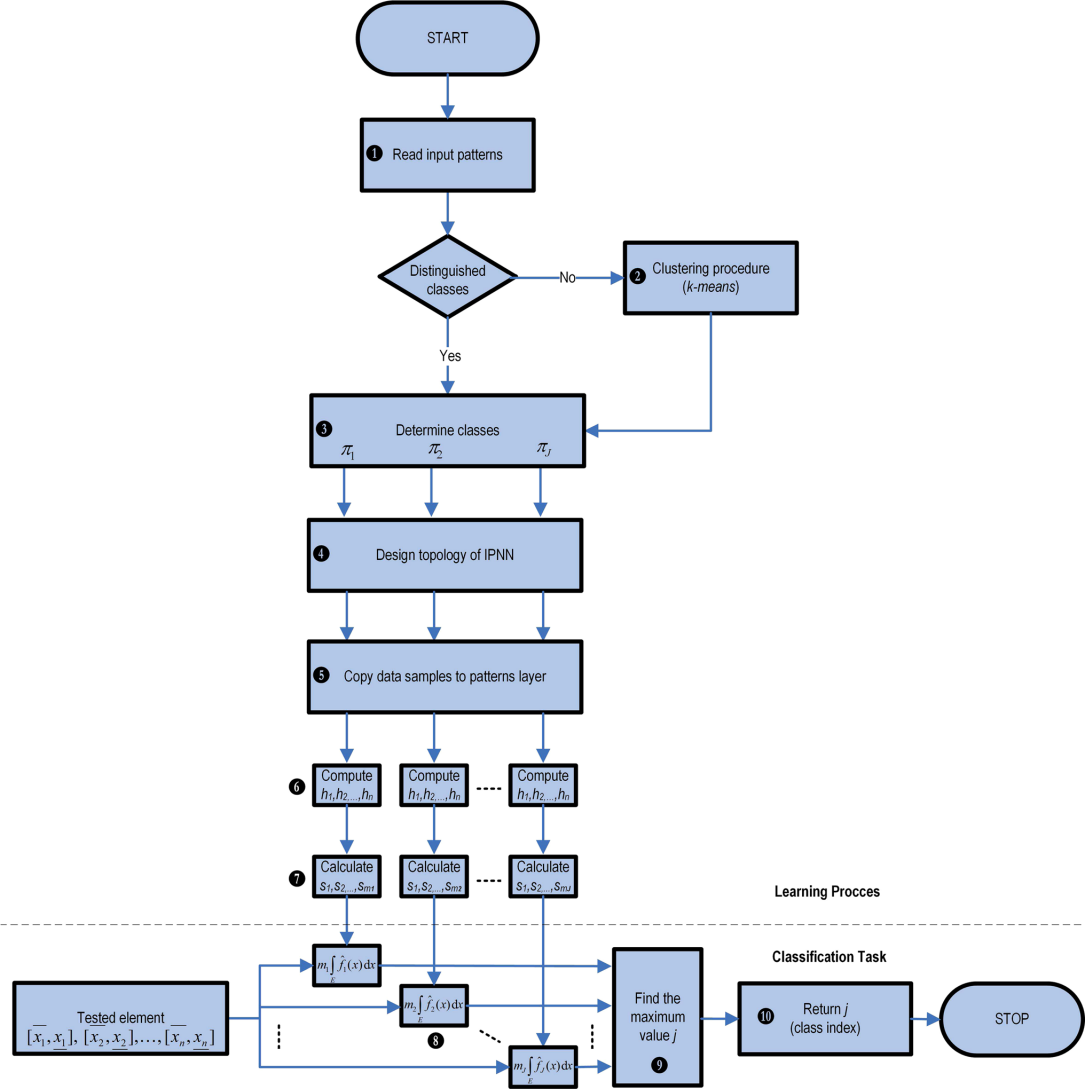
Flow chart of algorithm for the neural classification of interval information

In this section, a description is proffered of the entire network construction algorithm, as well as its learning and classification. All the components of the methodology detailed here are set out in Fig. 4. The diagram is, however, split into two parts: The upper part over the dashed line consists of a description of IPNN construction and related learning functions; the lower part reveals an application of IPNN for providing a solution for a particular classification task.

In the first part of the algorithm, we start from data acquisition ❶. If in the learning data set, particular classes are to be distinguished, it is advisable to proceed to step ❸; otherwise, necessarily, some algorithm for clustering the data has to be applied ❷. In a situation wherein the number of classes is a priori known, the application of simple *k-means* algorithm may be implemented. In a different situation, the clustering algorithm must be set out in addition to assigning the elements of the data set to individual classes. As these, above all, determine the number of classes under consideration, the Complete Gradient Algorithm (CGA) [17, 21, 22] is strongly recommended. A very big positive for utilizing this approach is the existing possibility of applying statistical kernel estimation methods—the same methodology as in the IPNN case. This latter algorithm has been successfully tested within many issues of business, science, and technology, for example, in developing marketing strategies, in modeling of dynamic control structures, and in image analysis. The main advantage of the CGA is that it does not require additional assumptions regarding the number of clusters, which allows it to better suit its obtaining number to real data structures [26].

Thus, we assume in step ❸ that the data that are to be distinguished are fully prepared so as to constitute the classifier. In the subsequent step ❹, it is necessary to design a neural network topological structure. In so doing, the number of classes considered, and the number of learning samples representing each class should be taken into account (a detailed description of ways to do so can be found in Sects. 2.4 and 3.1). After designing the structure of the network, it becomes necessary to copy all the training data into their corresponding neurons ❺ (which are found within the pattern layer, as described in Sect. 3.1).

In this way, we thus achieve the basic structure of a IPNN in which all parameters can be computed. As described in Sect. 2.2, for each of the classes, the algorithm for the smoothing parameter $h_i$ calculation is actuated. As a result of its use, *n* smoothing parameters (for each coordinate of the feature (input) vector *X*) are obtained ❻, taking the form of a vector $h_1, h_2, \dots , h_n$ with regard to the multidimensional case.

In the next stage ❼, we focus on designating inside each class, the *m* elements $s_j$. These constitute the modification of the smoothing parameters. This operation is carried out in accordance with the procedure described in Sect. 2.3. This step completes the procedure for the construction and learning of the IPNN.

In the following description of the algorithm, it is assumed that we intend to use the constructed neural network for classifying imprecise information. Therefore, the next set of characteristics set out will be related to the process of bringing about a neural classification that is intended for dealing with interval-type information. A detailed mathematical description has been placed in Sect. 3, while in Fig. 4, this is located under the dashed line.

A new tested element $\widetilde{x}\in {\mathbb {R}}$, consisting of imprecise information which is set out as an interval or interval vector, is levied from being a collection of items found throughout an input layer, to being a group of neurons inside a pattern layer. In each cell, the integration operation takes place in ❽, according to Eqs. (28)–(32) or (34)–(35) for the multidimensional case. This is followed up by the summation of signals from the previous layer, and taking into account the training set cardinality, a class distinction is made in ❾. The tested interval-type element then belongs to the class for which the output signal from the summation layer is the largest. The above setup is based on a generalization of the Bayes theorem for interval information and is described in the previous section. In this way, in ❿, the index (label) of the class according to formula (27) is obtained.

Taking into account ❽–❿, as well as the mathematical equations from section 3, this completes the application of IPNN for classifying imprecise information.

## Computational complexity of the IPNN algorithm

In order to fully describe this new algorithm, one of the more important properties from the point of view of modern computing will be discussed in this section. This is the computational complexity of time and memory. The estimation of these attributes allows a comparison of the described method with other established algorithms and provides an indication of the circumstances under which its use is appropriate.

Initially, time complexity will be presented. It is worth underlining the two-phased nature of the method presented in this paper. The first phase contains the procedures for constructing and learning the IPNN. Here, the most time-consuming element is applying the algorithm for calculating the smoothing parameter through the use of the plug-in method of complexity $O(nm^2)$ for every considered class. Similarly, this same complexity characterizes the calculations for the smoothing parameter modification procedure. At this point, it should be emphasized that these algorithms are executed once only, and this occurs at the beginning of the neural classifier procedure preparation.

The second phase consists of utilizing the algorithm for classifying imprecise information of an interval type. In contrast to the first phase, in this case, the procedure has linear time complexity with regard to all parameters *n*, *m,* and *J*, i.e., *O*(*nmJ*). This estimate implies the need for allotting a short calculation time for completing the classification task. Moreover, in providing solutions to encountered problems that are of a practical nature, it allows for the application of the IPNN methodology within real-time regime constraints. To facilitate a full understanding of the features of IPNN methodology, a short review of its memory complexity is required. This is related to an appreciation of the extent of work memory size as used when the IPNN algorithm is applied. In both steps of the presented procedure, this complexity has a linear characteristic with respect to all parameters (as mentioned in the previous discussion). Here, it should be emphasized that at the end of the learning phase, for each class, *n* smoothing parameters and *m* modification coefficients are stored within the algorithm. Thus, the complexity can be expressed as

36 $$\begin{aligned} J+\sum _{k=1}^{J}(m_k+n) \end{aligned}$$

of double precise memory elements. This is a very advantageous estimate that is a consequence of the lack of use of data structures such as lists, stacks, queues, and above all, the iterative, rather than recursive nature of the procedures employed.

## Numerical verification

Verification of the correctness of the IPNN presented in this paper when used for classifying interval information was conducted by way of numerical simulation. The following are the results of applications involving data obtained using a random number generator with normal distribution, and with a given vector of expected value, as well as a covariance matrix. It was derived from the implemented multivariate normal distribution generator, based on the concept of Box–Muller [4].

The quality assessment method as utilized in the initial part of the numerical verification is built upon data which were obtained by generating a set of random numbers for an assumed distribution, and then, an analysis was undertaken of the results correctness of the classification procedures for both the data types' interval, and by way of analogy, for an unambiguous number set. In this comparison case, a classification of precise data, the classical PNN was applied.

After obtaining the sequences of pseudorandom patterns representing the different classes, a test data set consisting of classified items was generated. This included interval types and, occasionally, unambiguous types (for comparative purposes). Each class corresponded to a set of size 1000 items.

The classified elements were obtained through generation, by utilizing one of the aforementioned generators set up by way of a normal distribution of the first pseudorandom number, with the second being taken from a generator with uniform distribution and which defines the location of the first as within an interval of arbitrarily assumed length. This represents information that is of interval type when there are no set circumstances for the considered imprecision, although its size is known. Such an interpretation seems to be the most appropriate for the majority of practical interval analysis applications. The tables below show the results as obtained by way of the following class cardinality (*m*): 10, 20, 50, 100, 200, 500, and 1000. In the mentioned tables, each cell contains the results gained through 100 tests, giving an average classification error that was defined on the basis of these 100 random samples.

The basic form of research conducted for the classification method developed here is built upon the information interval and on uniformly given patterns. For $n=1$, the data for the first class were generated using pseudorandom number generator setup with normal distribution *N*(0, 1), while the data of the second class with normal distribution *N*(2, 1). The results of this example are presented in Table 1. These results will provide comparative data in relation to that presented in the subsequent tables; therefore, this table may be deemed as being the basic example for comparative purposes. For comparison, in Table 1 and the other tables, a second column of results derived by way of employing a classic PNN classifier for precise data is shown.

**Table 1:** Results of the numerical verification in the case of pattern *N*(0, 1) *N* (2, 1)

*m*	Interval length						
	0.00	0.1	0.25	0.5	1.00	2.00	5.00
10	0.1713	0.1720	0.1720	0.1723	0.1729	0.1761	0.1944
20	0.1655	0.1669	0.1669	0.1672	0.1680	0.1713	0.1888
50	0.1602	0.1605	0.1606	0.1609	0.1617	0.1652	0.1848
100	0.1596	0.1601	0.1602	0.1604	0.1615	0.1650	0.1827
200	0.1596	0.1602	0.1604	0.1609	0.1618	0.1650	0.1840
500	0.1591	0.1595	0.1596	0.1602	0.1613	0.1647	0.1844
1000	0.1579	0.1584	0.1588	0.1591	0.1603	0.1637	0.1833

The neural classification—basic concept

In this table, in the last column (wherein the interval length is 5.0), we can see a rather curious phenomenon. Namely, it is in a sample size of 100 that we obtain the best result of the simulation. In our opinion, that this came about is associated with the choice of a rather extreme set of simulation parameters, as the length of the interval being equal to 5 is more the twice greater than the distance between the expected values of random samples of the examined classes. The aforementioned situation constitutes a rather random classification for a large number of cases.

### Multidimensional case

The case study presented here concerns the neural classification of multidimensional interval information. Generally, to demonstrate the transparency of the results in the tables presented in this article, three interval lengths will be contained. As a relatively small length of the interval a 0.1 was adopted, a further intermediate value of 0.5, being representative of the large length of 2.0, was then set.

First, the two-dimensional case will be considered. The patterns of classes were obtained using a pseudorandom number generator setup for normal distribution, with the expected values and unit covariance matrices. The results are shown in Table 2. It is worthwhile comparing these with the results of a one-dimensional base case as contained in Table 1, because this test case represents its two-dimensional equivalent.

The results obtained for the 50 elements sample are comparable with the outcome for the case of the one-dimensional sample with 10 members, and for patterns of stances, respectively, 100 and 20, 200 and 50, 500 and 100, as well as 1000 and 200.

**Table 2:** The results of the numerical verification for normal distributions with expected values $E_1= [0, 0]^{\rm T}$, $E_2= [2, 0]^{\rm T}$ and unit covariance matrices

*m*	Interval length		
	0.1 $\times$ 0.1	0.5 $\times$ 0.5	2.0 $\times$ 2.0
10	0.2082	0.2086	0.2109
20	0.1881	0.1882	0.1910
50	0.1725	0.1728	0.1762
100	0.1676	0.1677	0.1710
200	0.1630	0.1634	0.1679
500	0.1610	0.1615	0.1656
1000	0.1590	0.1593	0.1640

In Table 3, test results for the case of three-dimensional patterns of normal distribution, with the expected values and unit covariance matrices, are presented. Again, it is worthwhile comparing the results contained therein with the results shown in Tables 1 and 2, which were, respectively, obtained for the cases of one- and two-dimensional patterns.

**Table 3:** The results of the numerical verification for normal distributions with expected values $E_1= [0, 0, 0]^{\rm T}$, $E_2= [2, 0, 0]^{\rm T}$ and unit covariance matrices

*m*	Interval length		
	0.1 × 0.1 × 0.1	0.5 × 0.5 × 0.5	2.0 × 2.0 × 2.0
10	0.2295	0.2294	0.2302
20	0.2137	0.2140	0.2159
50	0.1906	0.1908	0.1911
100	0.1826	0.1824	0.1830
200	0.1760	0.1760	0.1767
500	0.1684	0.1687	0.1698
1000	0.1629	0.1631	0.1644

The results obtained for 200 elements sample size in three-dimensional case are comparable with outcomes obtained for 50 and 10 element sample sizes in two- and one-dimensional case, respectively. A similar situation exists for the samples of stances 500, 100, and 20, and 1000, 200, and 50.

The above relationships are direct demonstrations of the phenomenon of the “curse of dimensionality,” which is a consequence of the exponential increasing in the requirements for a random sample size allied with the increasing dimension of the task. It should be noted that in the case considered here, with regard to the issue of Bayesian classification of interval information, after ensuring the above requirements, the properties of the algorithm do not evoke the appended claims. Again, as in previous studies, with increasing sample size, as visualized in Tables 2 and 3, the average classification error diminishes. Additionally, while the length of the interval increases, then the value of misclassification expands. More numerical simulation concerning investigation on multidimensional data will be presented in Sect. 6.7, in which real multidimensional data are taken into account.

### Asymmetry of error for unbalanced pattern sets

Similar tests were performed as in the base case, for sets of classes of patterns that were generated by pseudorandom number generators for normal distributions *N*(0, 1) and *N*(2, 1). These tests reveal a symmetry of error in situations of evenly and unevenly matched patterns. Thus, we see in Table 4 the results for the case of a series of equinumerous patterns, while the subsequent two tables provide the results of classifications involving imbalanced patterns. In Table 5, the sample cardinality of the second class is twice larger than the first $(m_1 = 2m_2)$, while in Table 6, this value is ten $(m_1 = 10m_2)$. The number of misclassifications is presented here in two columns: The first is characterized—as before—by the total number of errors. In the second, the introduced column is divided into two rows, wherein the top contains an error based on the number of misclassified elements of the second class that are assigned to the first, while the bottom row is constituted of the error which arose from the number of first- class elements that are misclassified into the other.

Regarding the case of equinumerous patterning, as seen in Table 4, the average classification error does not show asymmetry, even at very low-class cardinality (i.e., $m=10$ or $m=20$). This situation, although generally consistent with intuition, is still worth noting.

With respect to cases of imbalanced data, the larger the pattern set generated, the greater the number of elements incorrectly classified (Table 5). This tendency is more visible as the ratio of sample cardinality is greater (Table 6). This is mainly due to the better quality of the pattern with increased cardinality.

It should again be noted that in each case (represented by each column of Tables 5 and 6), with increasing sample size, the average classification error gradually decreases. Moreover, along with the increase of the interval length, the value of the classification error increases.

**Table 4:** The numerical simulation results for equinumerous pattern sets, i.e., $m_1=m_2$

$m_2$	Interval length					
	0.1		0.5		2.0	
10	0.1720	0.0870	0.1723	0.0870	0.1761	0.0841
		0.0850		0.0853		0.0878
20	0.1669	0.0823	0.1672	0.0823	0.1713	0.0841
		0.0846		0.0849		0.0872
50	0.1605	0.0814	0.1609	0.0814	0.1652	0.0836
		0.0791		0.0795		0.0815
100	0.1601	0.0806	0.1604	0.0805	0.1650	0.0827
		0.0795		0.0799		0.0823
200	0.1602	0.0797	0.1609	0.0801	0.1650	0.0822
		0.0805		0.0808		0.0828
500	0.1595	0.0776	0.1602	0.0778	0.1647	0.0797
		0.0819		0.0824		0.0850
1000	0.1584	0.0769	0.1591	0.0770	0.1637	0.0784
		0.0814		0.0821		0.0853

**Table 5:** The numerical simulation results for imbalanced pattern sets, i.e., $m_1=2 m_2$

$m_2$	Interval length					
	0.1		0.5		2.0	
10	0.1703	0.096	0.1705	0.0958	0.174	0.0942
		0.0743		0.0747		0.0768
20	0.166	0.0923	0.1662	0.0924	0.1698	0.0942
		0.0737		0.0738		0.0756
50	0.1611	0.0896	0.1617	0.0898	0.1661	0.092
		0.0715		0.0719		0.0741
100	0.1605	0.0873	0.1608	0.0873	0.1649	0.0899
		0.0733		0.0734		0.075
200	0.1605	0.0874	0.161	0.0876	0.1658	0.09
		0.0731		0.0735		0.0758
500	0.1597	0.0862	0.1603	0.0862	0.1646	0.088
		0.0736		0.0741		0.0766
1000	0.1596	0.087	0.1599	0.0869	0.1641	0.0882
		0.0727		0.0731		0.0759

**Table 6:** The numerical simulation results for imbalanced pattern sets, i.e., $m_1= 10 m_2$

$m_2$	Interval length					
	0.1		0.5		2.0	
10	0.1753	0.1184	0.1759	0.1186	0.1791	0.1176
		0.0569		0.0572		0.0588
20	0.1718	0.1155	0.1722	0.1157	0.1754	0.1176
		0.0563		0.0565		0.0578
50	0.1691	0.1143	0.1697	0.1146	0.1740	0.1171
		0.0548		0.0552		0.0568
100	0.1675	0.1125	0.1682	0.1129	0.1723	0.1156
		0.0550		0.0553		0.0568
200	0.1680	0.1125	0.1684	0.1126	0.1726	0.1152
		0.0556		0.0557		0.0574
500	0.1665	0.1103	0.1671	0.1105	0.1717	0.1131
		0.0562		0.0566		0.0585
1000	0.1646	0.1100	0.1662	0.1095	0.1697	0.1126
		0.0546		0.0567		0.0571

### Multimodal pattern sets

The investigations herein are a continuation of the studies put forward in the previous section, but as applied to the multidimensional case and to classes consisting of patterns obtained by way of pseudorandom number generators for multimodal distributions. In the following test, the first class consists of a linear combination of three normal distributions of the expected values $E_{1,1}=[0,0]^{\rm T}$, $E_{1,2}=[2,0]^{\rm T}$ and $E_{3,1}=[4,0]^{\rm T}$, with unit covariance matrices and factors (coefficients) of the combination 1/3, 1/3, 1/3. A second pattern set contains a linear combination of the two normal distributions of expected values $E_{2,1}=[1,\sqrt{(3)}]^{\rm T}$, $E_{2,2}=[3,\sqrt{3}]^{\rm T}$, with unit covariance matrices and combinations ratios 1/2, 1/2.

These distributions are arranged in space so that their expected values are the vertices of an inverted letter W. The distances between the centers of adjacent patterns are also equal to 2, and this existence in the one-, two- and three-dimensional states is presented in Tables 1, 2, and 3, respectively. The results of this investigation (involving multimodal pattern data), as displayed in Table 7, are especially useful for a comparison with Table 2 for the two-dimensional case.

**Table 7:** Results of the numerical verification for a linear combination of normal distributions: the expected values $E_{1,1}=[0,0]^{\rm T}$, $E_{1,2}=[2,0]^{\rm T}$, $E_{3,1}=[4,0]^{\rm T}$ and unit standard deviations and factors of the combination 1/3, 1/3, 1/3 for the first class, and for the second class, with the expected values $E_{2,1}=[1,\sqrt{(3)}]^{\rm T}$, $E_{2,2}=[3,\sqrt{3}]^{\rm T}$ and unit standard deviations and factors of the combination 1/2, 1/2

*m*	Interval length		
	0.1 $\times$ 0.1	0.5 $\times$ 0.5	2.0 $\times$ 2.0
10	0.2438	0.2442	0.2509
20	0.2251	0.2257	0.2339
50	0.2067	0.2077	0.2166
100	0.1984	0.1992	0.2092
200	0.1929	0.1943	0.2041
500	0.1886	0.1899	0.2015
1000	0.1859	0.1869	0.1994

Regarding the multimodal pattern data (Table 7), the obtained results were about 10–15 % worse than those for the two-dimensional example (Table 2). This result is obvious from the intuitively point of view: Each of the modes for the individual pattern less accurate was estimated on the basis of a relatively smaller number of elements, and therefore, the patterns are naturally less accurate (np. for classification task) than those in the unimodal case.

However, apart from a slight deterioration in quality, the classification algorithm itself has not been amended in terms of its structure nor the speed of calculation. Such attributes are characteristic for nonparametric methods. Similar results were obtained for other more complex cases, both in terms of the number of modes and their mutual arrangement, also when the individual patterns consisted of mutually dislocated subsets separated using fragments of other patterns.

What is more, in this case, the average error of classification decreases with increasing cardinality of patterns, while expanding the interval length implies increasing the value of the classification error.

### Data *Toy 2D*

This subsection provides the reader with the results of the tests conducted to verify the appropriateness of IPNN in the classification benchmark data. However, due to the nature of current research in this field, with regard to this type data, in general, this data cannot be located within the available data repositories and web pages. Although there are several sets of interval data that can be found, for example, the oil [35] or the fish data set [32], these are designed to test only the clustering procedure and do not include reference elements that are given uniformly. Therefore, the interval data used in the following tests have been created on the basis of data obtained from repositories (with unambiguous data) in the same way as described at the beginning of the section.

The *Toy 2D* benchmark data set can be found at the following website [6]. It features a two-dimensional random sample—learning and testing—represented by way of the image of an eclipse of the Moon. The learning sets contained within consist of 2152 points associated with the first class, and 2444 points that are associated with the second. The test sample was drawn from the two-dimensional regular grid and includes 26,130 elements drawn from the first class and 34,371 drawn from the second.

**Table 8:** The results of the numerical verification for the data *Toy 2D*

Interval length	0.00 $\times$ 0.00	0.10 $\times$ 0.10	0.25 $\times$ 0.25	0.50 $\times$ 0.50	1.00 $\times$ 1.00	2.00 $\times$ 2.00
Classification error	0.0681	0.0737	0.0750	0.0766	0.0828	0.1097

Test results for classifying *Toy 2D* data are presented in Table 8. The results obtained in this classification test point to the fact that a neural algorithm copes well with inaccurate information. As is evident, an interval character, when introduced to the testing data, naturally engenders a deterioration in the test results. However, if we look at the range of these changes, we can see a difference varying from 1.7 to 8 % (each being evident in comparison with the results shown in the previous column). With regard to this, we consider the last result (for interval length 2.0$\times$2.0) as being incomparable due to having too high a degree of inaccuracy.

### Data *Synthetic Two-Class Problem*

In the following research, the data set was obtained from the well-known example described and referred to in the monograph of [33]. The data set *Synthetic Two-Class Problem* consists of two predefined subsets: pattern and testing for each of the two classes, and contains, respectively, 250 and 1000 differential elements.

The results of the use of this data set are seen in Table 9. These results show very similar conclusions to those presented in the previous sections. However, in the present case, the data range decreases significantly, resulting in, with respect to accuracy, the tested interval element significantly reducing the quality of the classification by the fifth column (that which corresponds to the length of 1.0).

**Table 9:** The results of the numerical verification for the data *Synthetic Two-Class Problem*

Interval length	0.00 $\times$ 0.00	0.10 $\times$ 0.10	0.25 $\times$ 0.25	0.50 $\times$ 0.50	1.00 $\times$ 1.00	2.00 $\times$ 2.00
Classification error	0.142	0.141	0.148	0.147	0.181	0.258

### Data *Iris Plant*

In this study, the collection of real data has been downloaded from a known repository located at the Center for Machine Learning and Intelligent Systems, at the University of California, Irvine, and made accessible on the web page [7]. This data set is built upon the measured length and width of both the petals and sepals of the flowers of three species of iris—*Setosa canadensis, versicolor*, and *virginica*. The first two classes are linearly separable. The data set consists of three equinumerous classes represented in total, by 150 items. However, the learning and testing samples are not distinguished. For this reason, in light of the study’s intentions, the data were randomly placed within two subsets containing patterns and testing elements with ratio coefficient 0.5. The results of this study are found in Table 10.

**Table 10:** The results of the numerical verification for the *Iris Data Set*

Interval length	0.00 $\times$ 0.00	0.10 $\times$ 0.10	0.25 $\times$ 0.25	0.50 $\times$ 0.50	1.00 $\times$ 1.00	2.00 $\times$ 2.00
Classification error	0.041	0.045	0.047	0.048	0.049	0.066

The table shows the average of 1000 bits of data placed within random divisions. The intervals were generated in the same manner as in the previous studies.

The results that were obtained highlight the many positive features of the neural classification method. The first is the minimal actual sensitivity to the “curse of dimensionality,” since the classification of a four-dimensional feature vector has been satisfactorily performed on the basis of pattern sets containing about 25 elements. Additional confirmation of the effectiveness of the method proposed herein was obtained by comparison with the results presented in [16] for unambiguous data. The above comparison revealed the presence of a classification error of no less than 4.5 %. A similar result was obtained in this study when working with unambiguous data (see the first column of Table 10). Despite the reduction in accuracy of classified information brought about by processing it into imprecise data, to the length of interval 1.0, the results have not deteriorated. This is worth underscoring.

### The data *Seeds Data set* and the data *Breast Cancer Wisconsin*

Further numerical study examples that illustrate the benefit of IPNNs are built upon the benchmark of real data collected under the names of Seeds Data set and Breast Cancer Wisconsin (Original). These data sets are located at the Center for Machine Learning and Intelligent Systems, at the University of California, Irvine, and made accessible on the web page [8]. Both sets of data are real data, nontrivial, and they are multidimensional. The obtained results indicate how the proposed neural network deal with multidimensional data.

The first data are drawn from images that were recorded on a $$13 \times 18$$ cm X-ray KODAK plate. Each data item consists of 7 coordinates derived from the geometric parameters of wheat kernels (*Triticum* spp.) These data include: area *A*, perimeter *P*, compactness $$C = 4*pi*A/P^2$$, length of kernel, width of kernel, asymmetry coefficient, and, finally, length of kernel groove. All these parameters are real-valued continuous. The examined group consisted of kernels belonging to three different varieties of wheat: Kama, Rosa, and Canadian. There are 70 elements in each class, randomly selected from an experiment exploration undertaken at the Institute of Agrophysics of the Polish Academy of Sciences in Lublin.

In this data set, as in the previous case, the learning and testing samples are not distinguished. For this reason, data were assigned to these groups with the ratio of 0.5. The results, shown in Table 11, demonstrate the average value of the error originating from 1000 repetitions of the classification test.

**Table 11:** The results of the numerical verification for the *Seeds Data set*

Interval length	0.00 $\times \cdots \times$ 0.00	0.10 $\times \cdots \times$ 0.10	0.25 $\times \cdots \times$ 0.25	0.50 $\times \cdots \times$ 0.50	1.00 $\times \cdots \times$ 1.00	2.00 $\times \cdots \times$ 2.00
Classification error	0.0777	0.0772	0.0798	0.0828	0.0869	0.1018

The next data set is built upon breast cancer databases obtained from the University of Wisconsin Hospitals, Madison, and compiled by Dr. William H. Wolberg [27]. This data set contains 683 instances of samples, described using 10 features (i.e., $n=10$). These data are the genuine case histories of medical research cancer patients. Benign cancer patients are described in the first class, by way of using 444 data examples, while patients with advanced malignant cancers make up the second group of data of 239 samples. For the purposes of classification, the data were divided into learning data and testing group, in a proportion of 0.5. All tests were performed 1000 times, and the mean value of the classification error is shown in Table 12.

**Table 12:** The results of the numerical verification for the *Breast Cancer Wisconsin*

Interval length	0.00 $\times \cdots \times$ 0.00	0.10 $\times \cdots \times$ 0.10	0.25 $\times \cdots \times$ 0.25	0.50 $\times \cdots \times$ 0.50	1.00 $\times \cdots \times$ 1.00	2.00 $\times \cdots \times$ 2.00
Classification error	0.0296	0.0301	0.0365	0.0404	0.0486	0.0692

By analyzing the data obtained from the numerical verification of the Seeds Data set, a very interesting phenomenon is noticeable. In the second column, the classification error value decreases when compared to the previous column. The reduction in the classification error for the data (which is affected by inaccuracy derived from the interval) is unnatural, because the decline in the quality of data should result in an increase of misclassification. In this case, the phenomenon occurs only for the data represented by the smallest interval. It can be assumed that positive classification result, which was achieved in this case, is due to the averaging character of the integration operator used for the KDE method.

It can be additionally concluded that this problem is solvable, but with a relatively small decline in both the quality of classification and the quality of the information represented by the length of the interval. Both examples, when compared to the other algorithms, however, again show the superior quality of the proposed neural network in overcoming the “curse of dimensionality.” This fact particularly refers to robustness of the classification algorithm in the case of the growing accuracy of the interval data.

### Comparison with similar algorithms for classification

Inaccurate information being represented by interval data is well known in scientific research. Indeed, presently, there are many algorithms that can deal with this type of data. As a first example, let us recall the procedure for a decision tree based on the Fuzzy Neural Network that is put forward by [42]. This paper presents a concurrent Fuzzy Neural Network approach to a developed decision support system for dealing with imprecise information which is built upon the Fuzzy C-Means Clustering procedure. Other papers that are devoted to interval-valued fuzzy decision tree methods are proposed in [24] [25]. In these cases, the model for the contracting decision tree, in using interval-valued fuzzy membership values, employs look-ahead based fuzzy decision tree induction and interval-valued fuzzy sets. This approach is useful in situations wherein ascertaining the precise values of the fuzzy membership parameters is not possible.

Another example of the use of interval types of information is contained in [15]. Herein, the authors propose a classification algorithm based on inaccurate pattern sets. However, in this case, the cited algorithm has different assumptions, since the imprecise reference data are of the interval type, what is in opposition to the IPNN method, where precise data type reference data are used.

The last to be listed here is the method associated with Interval Computing in Neural Networks [31]. In this case, authors propose constructing one-layer interval neural networks for processing imprecise-valued information. In this method, interval input data (including the training data) and interval-valued weights are applied.

As can be seen in the above brief review, there are various methods employing the information interval notion in dealing with raw data. However, current methods either cannot be applied to the classification task, or those that are dedicated to a classification problem are built upon different assumptions with respect to the method described in this article. Here it is good to recall once more that the proposed procedure is used for classifying imprecise information represented by the interval vector when the patterns of particular classes are given as sets of precise data elements. The subject of this subsection is a comparison of the quality of classification of inaccurate information of the interval type done by way of the method developed in this work, with two others: one currently available in the literature and one which is of natural character. The results of numerical verification based on these methods are shown in Tables 13 and 14. The first group of data (Table 13) was derived by way of employing Support Vectors Machine (SVM), a methodology broadly used today (due to its particular advantages), while the other was derived by comparing the number of elements of each pattern contained in the investigated interval (Table 14). The results shown in Table 13 were compiled by way of using the SVM procedure, in accordance with the algorithm presented in the work [45]. As a result of this procedure, there are three types of decisions: the assignment of an interval element to the first or to the second class, or the lack of this assignment.

**Table 13:** The results of verification by way of using SVM for normal distributions *N*(0, 1) and *N*(2, 1)

*m*	Interval length								
	0.1			0.5			2.0		
	Error	No decision	Full error	Error	No decision	Full error	Error	No decision	Full error
10	0.2202	0.0460	0.2432	0.1478	0.1892	0.2424	0.0989	0.3197	0.2588
20	0.2020	0.0652	0.2346	0.1166	0.2355	0.2343	0.0965	0.3146	0.2537
50	0.1678	0.0437	0.1897	0.1152	0.1518	0.1911	0.0789	0.2799	0.2189
100	0.1572	0.0225	0.1685	0.1243	0.0920	0.1703	0.0698	0.2666	0.2031
200	0.1538	0.0148	0.1612	0.1273	0.0692	0.1620	0.0669	0.2582	0.1960
500	0.1530	0.0129	0.1595	0.1286	0.0627	0.1599	0.0646	0.2587	0.1940

The study considered the amount of misclassification, lack of decision, and the total error. This last is the sum of bad decisions and those of the elements for which no decision was made as to placement. The latter were classified by drawing lots in a relation that is proportional to the numbers of patterns (for the case considered here, equinumerous patterns in the proportion of 0.5 and 0.5).

Comparing the above results with the ones revealed in Table 1 (the basic case), it is clear that the outcomes obtained using the SVM method are worse by 5 up to 50 %. If, however, admitting that the failure of the decision is the correct action, the above argument ceases to be absolutely true, yet it is still evident that the obtained results are different after all the conditions of the problem are considered.

The second, relatively simple, method of classifying interval-type information is the procedure for pattern counting. This consists of reckoning how many elements of the learning sample are contained in the interval which is under consideration. In this algorithm, the results obtained were reported by way of four cases: one incorporating the amount of miscalculation; one in which the value is derived from a situation wherein the number of elements from both patterns belonging to the tested element of the interval type is equal; one in which the subsequent amount describes a case wherein the inaccuracy elements do not contain any learning data; and, finally, one which encompasses the total error. This last is the sum of wrong decisions, and it also incorporates a resulting erroneous draw ratio of 0.5 and 0.5 for both cases: wherein the number of elements of both samples classes was the same, as well as when the tested element of the interval type does not contain any learning data. The numerical verification of all these situations is presented in Table 14.

**Table 14:** The results of verification by way of counting the elements contained in the interval pattern data for normal distributions *N*(0, 1) and *N*(2, 1)

*m*	Interval length											
	0.1				0.5				2.0			
	Error	Equal el.	No el.	Full error	Error	Equal el.	No el.	Full error	Error	Equal el.	No el.	Full error
10	0.0216	0.0017	0.9086	0.4768	0.0751	0.0223	0.6440	0.4083	0.1296	0.0573	0.2761	0.2962
20	0.0400	0.0055	0.8291	0.4572	0.1063	0.0435	0.4546	0.3553	0.1469	0.0483	0.1558	0.2489
50	0.0775	0.0227	0.6461	0.4120	0.1345	0.0555	0.2377	0.2810	0.1543	0.0296	0.0722	0.2052
100	0.1085	0.0426	0.4592	0.3594	0.1474	0.0422	0.1297	0.2333	0.1588	0.0177	0.0387	0.1870
200	0.1299	0.0557	0.2834	0.2994	0.1525	0.0272	0.0717	0.2020	0.1617	0.0104	0.0211	0.1774
500	0.1473	0.0454	0.1313	0.2356	0.1563	0.0152	0.0319	0.1799	0.1632	0.0048	0.0093	0.1703

The results gathered from the use of the concept of counting can be seen as decidedly worse than those obtained using the method developed in this article. As it is evident in a side-by-side comparison, these results are comparable only with results obtained for large set of samples (i.e., $m=500$) and with long interval elements (interval length $= 0.5$ or 2.0).

## Conclusions

This paper proposes a new type of neural network, the interval probabilistic neural network, which is a generalization of the established PNN for processing inaccuracy information of the interval type. In our study, IPNN was applied to the classification of multidimensional imprecise information of the interval type, where patterns of particular classes are given on the basis of sets of precisely defined elements. The presented methodology is based on the generalization of Bayes theorem for processing inaccuracy information.

This study reveals in detail the topological structure of an IPNN, as well as the whole process of learning that is involved, with particular attention placed upon the method used for calculating the smoothing parameter (the plug-in algorithm) and the procedure for modifying this. In the proposed topological structure, the form of the neurons in the pattern layer is modified by replacing the radial transfer functions with an integral operator. Moreover, in the summation layer, the neurons are enriched by way of employing normalization operations. In addition, the methodology applied here takes into account the cardinality of the patterns set in each class. The numerical studies contained within this article fully confirmed the positive properties of the presented method. Of note, the research was conducted using both random and real benchmark data. In particular, the results indicate that the developed neural classification algorithm can be successfully applied to data derived from complex pattern classes which are inseparable, multimodal, or even consisting of separate subsets deposited alternately. To summarize the results presented in the previous section, we can conclude that these have confirmed the correctness of the neural algorithm that is the subject of this paper, and which is developed here for the purpose of classifying inaccurate information. The obtained numerical results were compared with those achieved when the tested element has an unambiguous character by the way of classical PNN application. Furthermore, the achieved results were also compared with the outcomes obtained by the SVM procedure and by the method which counts the elements of data samples which are contained inside a classified interval element. The analysis performed here indicates that in the above procedures, a significant difference is evident in the results given as error based on the number of misclassifications for each length of interval. In the investigated procedure that is the subject of this article, this effect was significantly reduced by averaging the properties of nonparametric estimation included in the interval probabilistic neural networks. By using nonparametric statistical methodology, the independence of the IPNN procedure was affected from the occurring distribution of the data. This, in practice, is important especially in the case of nonstandard and multimodal patterns. Additionally, it should be noted that the presented neural network can be naturally generalized so as to deal with the case of multidimensionality. On testing the classifier based on IPNN, certain general conclusions can be observed. It can be seen that the loss of information resulting from the introduction of imprecision that is of an interval type did not cause a significant increase in error of classification (carried out here also using the method investigated in this paper) in the first columns of Tables 1, 8, 9, 10, 11, and 12 of numerical results, corresponding to the interval lengths 0.1, 0.25, 0.5, and 1.0. However, for the lengths 2.0 and 5.0, where they are the multiples of the range of sample data, such imprecise interval information obviously considerably lowers the quality of classification. However, in all studies, an expansion of the cardinality of pattern sets resulted in reducing the average value of misclassification. In practice, this allows a successive improvement of the quality of the classification as new data are acquired. It should be noted that due to the characteristics of the described method, it is possible to break the neural process into two parts. With regard to this, the time-consuming algorithms for calculating the required parameters and modeling topology of the neural network are included in the first phase, while the second phase consists of determining the class membership of the new tested element. This last action is based mainly on the direct application of analytical models and can be performed in a relatively short period of time, and through an online regime. The proposed interval neural network is a response to the increasing interest in the processing of data that is of an imprecise character. The issue of information classification on the basis of interval data can be illustratively interpreted when unambiguous examples of the patterns are composed of specific, precisely measured data, while the compartments represent limitations in the plans, or estimates, or when their measurements are difficult to perform. In particular, this neural method can be used for a classification where unambiguous data are treated as being specific information from the past (for example, temperature or exchange rates), while the classified element represents the inaccuracies that are forecasted in a manner that is naturally limiting.
